# How Does a Delay Between Temperate Running Exercise and Hot-Water Immersion Alter the Acute Thermoregulatory Response and Heat-Load?

**DOI:** 10.3389/fphys.2019.01381

**Published:** 2019-11-22

**Authors:** Storme L. Heathcote, Peter Hassmén, Shi Zhou, Lee Taylor, Christopher J. Stevens

**Affiliations:** ^1^School of Health and Human Sciences, Southern Cross University, Coffs Harbour, NSW, Australia; ^2^Laboratory for Athlete Development, Experience and Performance, Southern Cross University, Coffs Harbour, NSW, Australia; ^3^School of Sport, Exercise and Health Sciences, Loughborough University, Loughborough, United Kingdom; ^4^Faculty of Health, Sport and Exercise Discipline Group, University of Technology Sydney, Sydney, NSW, Australia; ^5^Human Performance Research Centre, University of Technology Sydney, Sydney, NSW, Australia; ^6^ASPETAR, Qatar Orthopaedic and Sports Medicine Hospital, Athlete Health and Performance Research Centre, Doha, Qatar

**Keywords:** heat acclimation, heat stress, hot bath, passive heating, endurance athletes

## Abstract

Hot-water immersion following exercise in a temperate environment can elicit heat acclimation in endurance-trained individuals. However, a delay between exercise cessation and immersion is likely a common occurrence in practice. Precisely how such a delay potentially alters hot-water immersion mediated acute physiological responses (e.g., total heat-load) remains unexplored. Such data would aid in optimizing prescription of post-exercise hot-water immersion in cool environments, relative to heat acclimation goals. Twelve male recreational runners (mean ± SD; age: 38 ± 13 years, height: 180 ± 7 cm, body mass: 81 ± 13.7 kg, body fat: 13.9 ± 3.5%) completed three separate 40-min treadmill runs (18°C), followed by either a 10 min (10M), 1 h (1H), or 8 h (8H) delay, prior to a 30-min hot-water immersion (39°C), with a randomized crossover design. Core and skin temperatures, heart rate, sweat, and perceptual responses were measured across the trials. Mean core temperature during immersion was significantly lower in 1H (37.39 ± 0.30°C) compared to 10M (37.83 ± 0.24°C; *p* = 0.0032) and 8H (37.74 ± 0.19°C; *p* = 0.0140). Mean skin temperature was significantly higher in 8H (32.70 ± 0.41°C) compared to 10M (31.93 ± 0.60°C; *p* = 0.0042) at the end of the hot-water immersion. Mean and maximal heart rates were also higher during immersion in 10M compared to 1H and 8H (*p* < 0.05), despite no significant differences in the sweat or perceptual responses. The shortest delay between exercise and immersion (10M) provoked the greatest heat-load during immersion. However, performing the hot-water immersion in the afternoon (8H), which coincided with peak circadian body temperature, provided a larger heat-load stimulus than the 1 h delay (1H).

## Introduction

Exercise in a hot environment increases thermoregulatory and physiological strain (Cheuvront et al., 2010) and unpleasant thermal perceptions (Kamon et al., 1974; Stevens et al., 2018), which contribute to deteriorated performances (Guy et al., 2015). Considering that many major sporting events are held under hot and humid conditions, including the upcoming Tokyo 2020 Summer Olympic Games (Kakamu et al., 2017), endurance athletes are recommended to employ heat acclimatization (training in natural heat) or heat acclimation (training in artificial heat) strategies (both abbreviated to HA) to negate heat-mediated performance decrements and possibly provide some protection against exertional heat illnesses (Racinais et al., 2015; Kakamu et al., 2017). Factors central for HA are increased sweating, and elevated core temperature (Tc) and skin temperature responses (Wendt et al., 2007; Périard et al., 2015; Neal et al., 2016). As such, acute HA training sessions aim to maximize these responses.

A mean performance improvement from HA programs of 7 ± 7% has been demonstrated across 27 datasets, where 24/27 reported an improvement >1% (Tyler et al., 2016). Strategies to implement HA into a program prior to a major competition contingent to travel circumstances are also available (Saunders et al., 2019). As such, the positive benefits on performance and recommendations for implementation of HA are clear, yet evidence-based active HA protocols (typically involving specialized facilities and/or relocation) may be logistically difficult to incorporate into complex training programs and the schedules of elite athletes (Casadio et al., 2017). The training sessions themselves can also be onerous, generally involving exercise in the heat for 30–100 min, preferably on consecutive days, for a minimum duration of 1 week (Houmard et al., 1990; Périard et al., 2015; Casadio et al., 2017). Despite the ergogenic potential, during the International Association of Athletics Federations (IAAF) World Athletics Championships in Beijing 2015, where hot and humid conditions were predicted, only 15% of athletes engaged in HA prior to competition (Périard et al., 2017), suggesting that implementing HA may be challenging for some athletes.

In response to these challenges, alternative HA strategies have been investigated, including post-exercise sauna bathing (Scoon et al., 2007) and post-exercise hot-water immersion [HWI; (Zurawlew et al., 2016)]. A total of 16 original investigations have been performed on the topic to-date (Heathcote et al., 2018); the majority demonstrating beneficial hallmark physiological adaptations of heat acclimation (including lowered resting and exercising Tc and heart rate, and increased plasma volume), and importantly, these adaptations were demonstrated in both recreationally active and endurance-trained individuals (Zurawlew et al., 2018a). Further, the use of post-exercise sauna (12 × 30 min exposures) improved running time to exhaustion by 32% in competitive runners/triathletes (Scoon et al., 2007) and post-exercise HWI (6 × 40 min exposures) improved 5 km running performance time in the heat by 4.9% in recreationally active individuals (Zurawlew et al., 2016).

Post-exercise HWI therefore presents a practical HA strategy for athletes residing in cooler climates, compared to expensive alternatives requiring artificial heat chambers and/or relocation. Passive heating has typically been applied immediately after exercise training when used for HA purposes (Scoon et al., 2007; Stanley et al., 2015; Zurawlew et al., 2016), with exercise conducted in laboratory settings, enabling easy access to heating facilities. Practically however, the ability to commence HWI immediately after exercise could be challenging for athletes who lack such facilities near training locations. Indeed, a delay of up to 1 h between training and HWI could easily occur when considering the activities that may prevent immediate immersion (e.g., debrief with coach, stretching, travel, bath preparation, etc.). In other circumstances, athletes may have other commitments during the day, which could delay HWI until the afternoon/evening. Precisely how such a delay and the observed Tc circadian oscillation across a day interact to potentially alter HWI-mediated physiological responses (e.g., total heat-load) remains relatively unexplored. Therefore, the aim of this study was to assess the acute physiological responses central to thermoregulatory strain (Tc, skin temperatures, heart rate, and sweat rate) when post-exercise HWI (30 min; 39°C) was delayed for 10 min (10M), 1 h (1H), or 8 h (8H) following a temperate treadmill run (18°C). It was hypothesized that both 1 and 8 h delay between exercise and HWI would reduce the thermo-physiological strain (e.g., heat-load) of the HA session.

## Materials and Methods

### Participants

Twelve male, recreational [i.e., performance level one-two (De Pauw et al., 2013)] long distance runners (mean ± SD; age: 38 ± 13 years, height: 180 ± 7 cm, body mass: 81 ± 13.7 kg, body fat: 13.9 ± 3.5%) volunteered for the study. Females were excluded due to the confounding influence of menstrual cycle mediated Tc fluctuations (Wendt et al., 2007; Mee et al., 2017). Inclusion criteria stipulated that the participants had performed a 10 km time trial within 6 months prior to the study in ≤50 min (mean time: 47 ± 3 min, range: 42–49 min). Exclusion criteria included any contraindications to exercise as per the Exercise and Sports Science Australia adult pre-exercise screening tool, previous diagnosis of low blood pressure, history of heat illness, or use of prescribed medication during the time of the study. Approval for the project was granted by the Human Research Ethics Committee at Southern Cross University (Approval number: ECN-17-121), and written informed consent was obtained before commencing any testing procedures.

### Experimental Design

**Figure 1:**
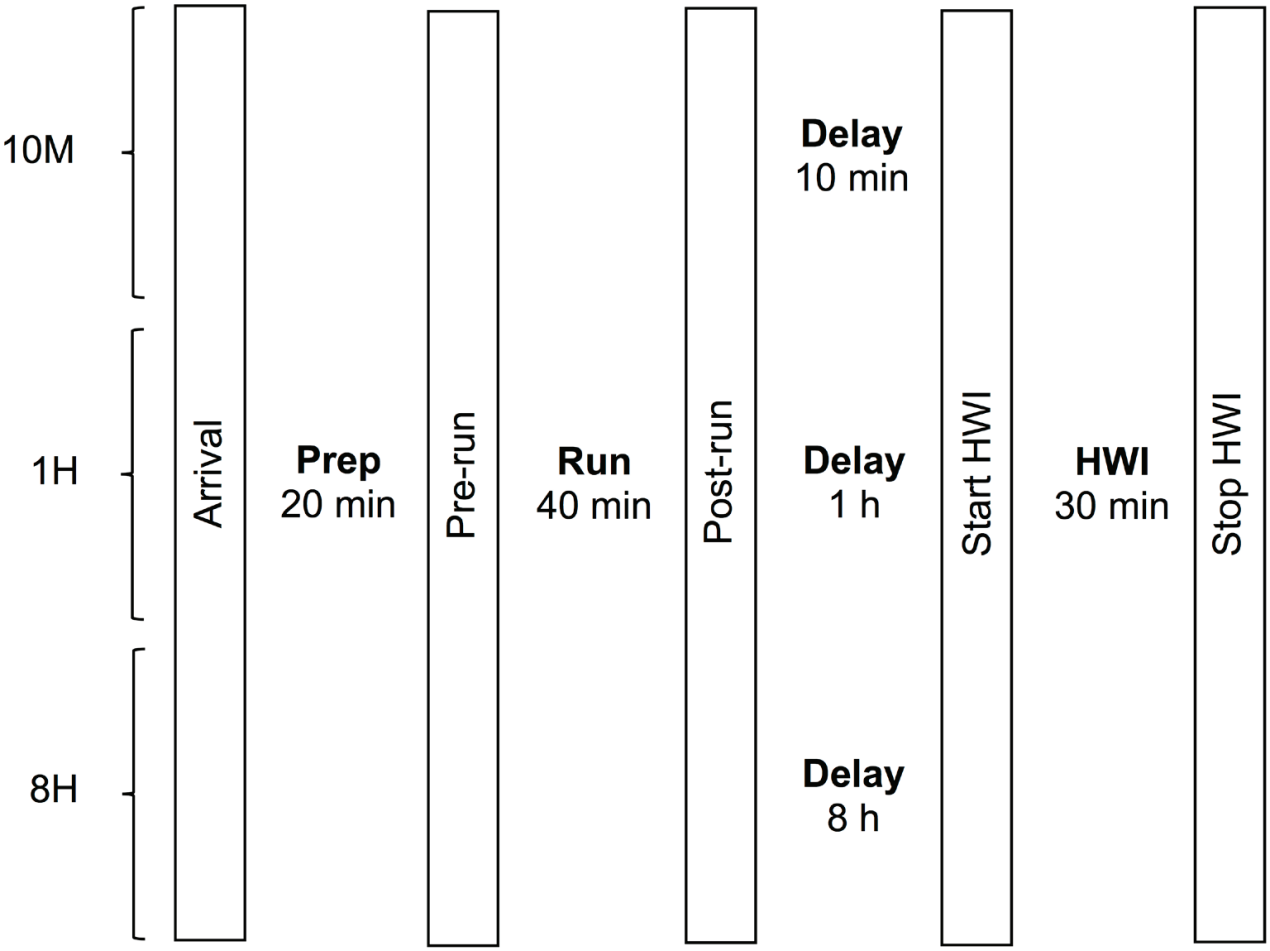
Schematic of the experimental design. 10M, 10 min delay; 1H, 1 h delay; 8H, 8 h delay; HWI, hot-water immersion.

Participants completed a 40-min submaximal treadmill run (Trackmaster TMX425 CP, Carrollton, Texas, USA) before a 30-min bout of HWI, on three separate occasions, 7–10 days apart. With a randomized crossover design, each trial involved a different time delay between exercise and HWI, including 10 min (10M), 1 h (1H), and 8 h (8H). A schematic of the experimental design is illustrated in Figure 1. During 8H, participants were permitted to leave the laboratory after the run and conduct their normal daily activities but were instructed to avoid any physical activity (all participants confirmed that they complied with these instructions). Participants were required to avoid alcohol and caffeine during testing days and to ensure adequate hydration by ingesting 500 ml of water 2 h prior to arrival. Data collection was completed during winter to minimize natural HA. The data collection took place in the Northern Rivers Region of NSW, Australia. The participants generally arrived to the laboratory wearing a tracksuit and they all ran in shorts and a short sleeve top.

### Exercise Protocol

A 40-min treadmill run [climate controlled laboratory; 18.0 ± 0.9°C; relative humidity (RH) 64.5 ± 4.7%] commenced in the morning (between 06:00 and 07:30; time held consistent after first laboratory visit), to control for circadian variation of internal body temperature (Słomko and Zalewski, 2016). A pedestal fan set at a wind speed of 10 km^.^h^−1^ was placed 2.5 m in front of the treadmill to replicate the convective cooling of running outdoors. During the first trial, running speeds were self-selected *via* rating of perceived exertion (RPE) (Borg, 1998). Participants were instructed to run for 10 min at “light” intensity (RPE = 11), 20 min at “hard” intensity (RPE = 15), and further 10 min at “light” intensity. The treadmill speeds were recorded and replicated in subsequent trials so that each participant ran at the same speeds in all trials. Participants consumed water at 33°C *ad libitum* during the run. This temperature was chosen to minimize any cooling effect from the fluid on the ingested capsule while remaining palatable.

### Hot-Water Immersion

The HWI strategy used was 30 min at 38.9 ± 0.1°C to the level of the waist wearing shorts. This was implemented using a bathtub (2.3 m long × 1.1 m wide × 0.5 m high) in a bathroom (8 m^2^; 24.2 ± 2.3°C, 76.3 ± 8.1% RH), with water temperature and flow maintained using a two-tap mechanism. Consumption of fluids during immersion was not permitted. The strategy used was based on piloting that determined 39°C was the highest temperature that was safe for the participants to complete with the specified depth and duration in the environment available (i.e., a small room with high humidity; representing the average bathroom). We note that this HWI strategy is less aggressive than that investigated previously (i.e., 40°C for 40 min to the level of the neck), which was too demanding for 6/10 participants to complete on the first exposure in the previous study (Zurawlew et al., 2016). Hence, the HWI strategy presented here is designed to be safe and achievable for the first exposure, and the demands of the HWI (i.e., increased temperature, depth, and/or duration) may be increased toward 40°C for 40 min to the level of the neck in subsequent exposures over time as appropriate for the individual.

Immersion termination criteria was set according to ethical requirements (i.e., reaching a Tc >39.4°C, rapid increase in heart rate, light headedness or reporting a thermal comfort rating that reached “very uncomfortable”); however, all participants completed the full 30-min protocol.

### Measurements

Before each initial experimental trial, participants underwent anthropometrical measurements including body mass by an electronic scale (Charder MS3200, Taichung City 412, Taiwan), stature (S+M Height Measure 2 m, Rosepark, SA) and skin-fold measurement by caliper (Harpenden Calipers, Baty International, West Sussex, United Kingdom) at seven sites including the bicep, tricep, subscapular, supraspinalae, abdominal, mid-thigh, and medial calf, following the International Society for the Advancement of Kinanthropometry recommended protocol (Marfell-Jones et al., 2012).

The Tc was measured continuously using an e-Celsius ingestible telemetric capsule (BodyCap, Caen, France). Participants were instructed to ingest the capsule with water immediately prior to sleep the night before each trial (approximately 8 h prior to each trial). Measurements of skin temperature were taken using a dermal thermal scanner (DermaTemp, Exergen Corporation, MA, USA) on dry skin at four sites (forehead, right calf, right hand, and lower back) before and after exercise/immersion, which allowed for an estimate of mean skin temperature (Tsk) according to the following equation (Nielsen and Nielsen, 1984):

Tsk=9.429+0.137×foreheadtemp+0.102×handtemp+0.29×backtemp+0.173×calftemp

Resting body temperature measurements occurred in the climate-controlled laboratory described above (18°C, 65% RH). All participants sat in the climate controlled laboratory wearing exercise clothing for a period of 20 min prior to exercise in all trials. During 10M and 8H, participants spent 10 min in the climate-controlled laboratory wearing shorts only immediately prior to immersion. In 1H, participants spent the entire 60 min in the climate-controlled laboratory prior to immersion; they wore clothing that allowed them to feel comfortable for 50 min, and then shorts only for the final 10 min.

Nude, dry body mass (accurate to 10 g) was recorded prior to the run (equipment same as above) after emptying the bladder. Body mass was also recorded prior to and after immersion following the same procedure. Water bottles were also weighed before and after the exercise to calculate fluid intake during the run so that sweat rate was estimated with the following equation;

SweatrateSR=changeinbodymass+fluidingested/time/bodymassinitial

Heart rate responses were measured continuously using a Garmin Forerunner 920XT heart rate monitor (Garmin, Neuhausen am Rheinfall, Switzerland).

Measurements of thermal comfort and thermal sensation were recorded at 5 min intervals during exercise and immersion. A four-point scale (1–4) was used to assess thermal comfort (Gagge et al., 1967) and a 17-point scale (0.0–8.0) was used to assess thermal sensation (Young et al., 1987).

### Statistical Analyses

The data were analyzed with General Linear Mixed Models and Tukey *post hoc* tests with multiplicity adjusted *p*’s (significance level = 0.05) using GraphPad Prism version 8.0.0 (GraphPad Software, San Diego, California USA). Visual inspection of residual plots did not reveal any obvious deviations from homoscedasticity or normality. Results are reported as mean ± standard deviation (SD). The magnitudes of any differences between conditions were expressed as standardized differences (effect sizes; ES). The criteria used for interpreting the magnitude of the ES were: ≤0.2 (trivial), >0.2 (small), >0.6 (moderate), >1.2 (large); and > 2.0 (very large) (Hopkins et al., 2009). The ES are reported with uncertainty of the estimates shown as ±90% confidence limits (CL). If the CL crossed both positive and negative trivial ES values, the magnitude was deemed unclear (Hopkins et al., 2009). A sample size calculation was performed (G*Power 3.1.2) with alpha level set at 0.05 and power set at 0.8, which revealed a sample size of nine participants was required to detect a meaningful change in Tc (0.3°C; Zurawlew et al., 2016).

## Results

There were no significant differences between conditions for any measured variable during the treadmill runs (*p* > 0.05). The mean treadmill speeds throughout the three running components of the trials were 9.4 ± 0.9, 12.4 ± 1.2, and 9.4 ± 0.9 km^.^h^−1^. When all trials were combined, the mean maximum heart rate during the run was 153 ± 12 bpm, and the mean sweat rate during the treadmill run was 0.20 ± 0.03 ml^.^min^.^kg^−1^. There were no significant differences between conditions for temperature of the water during HWI (*p* > 0.05).

**Figure 2:**
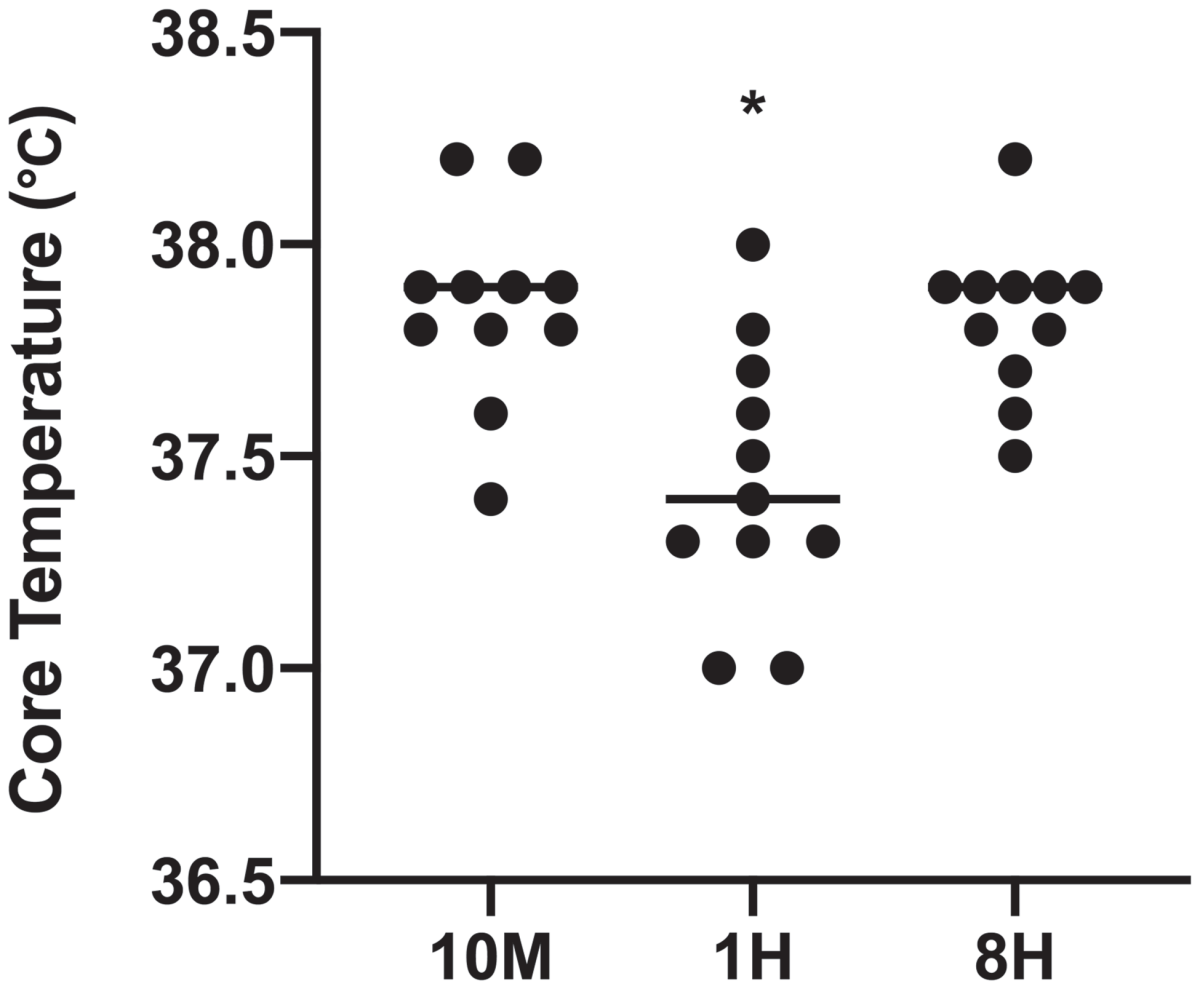
Mean and individual core temperature during hot-water immersion for each condition; 10 min (10M), 1 h (1H), and 8 h (8H) intervals (*n* = 11). ^*^Significantly different to 10M and 8H. Lines represent the mean and circles represent the individual responses.

**Figure 3:**
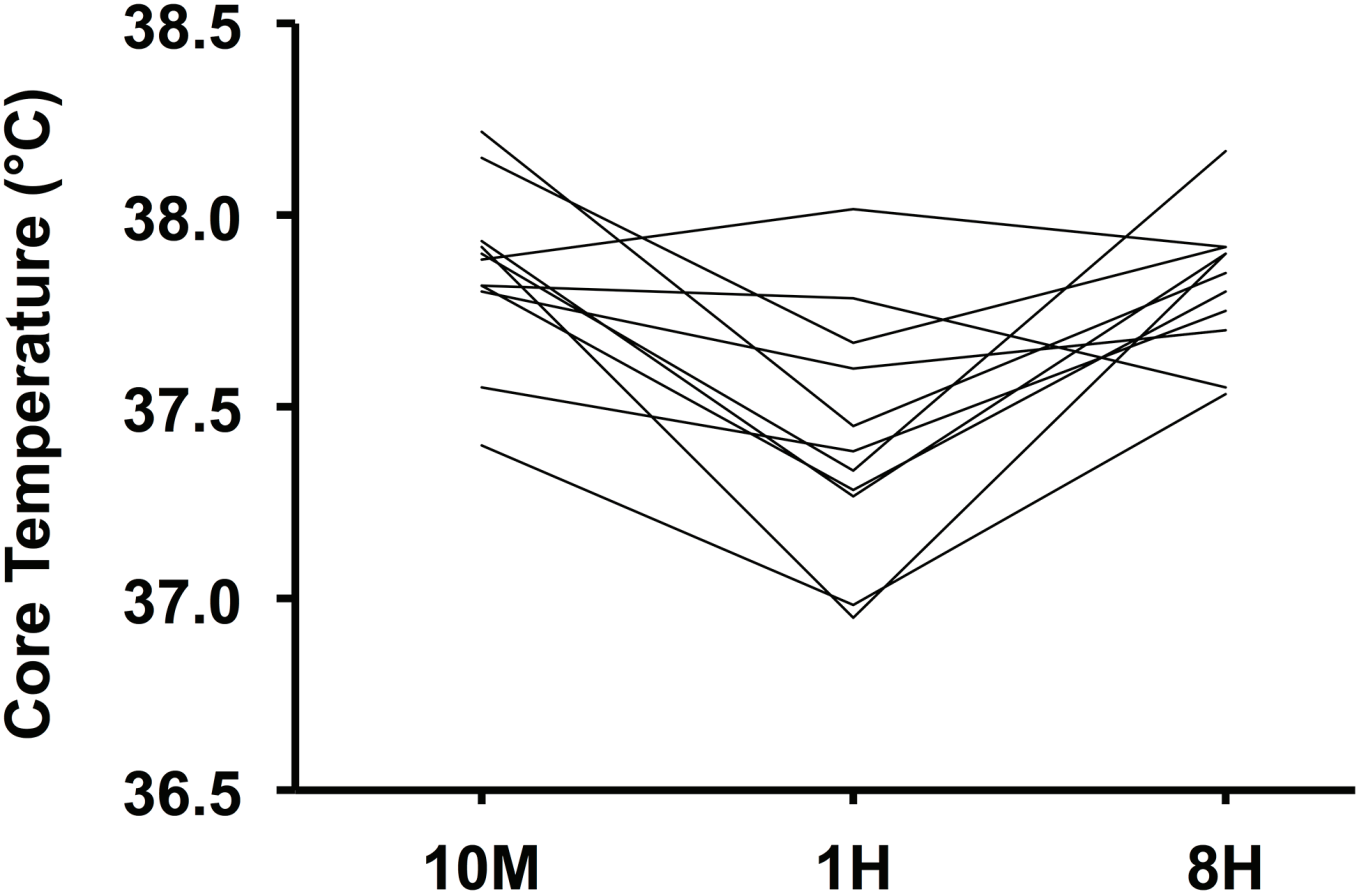
Trends in individual mean core temperature during hot-water immersion across conditions; 10 min (10M), 1 h (1H), and 8 h (8H) intervals (*n* = 11).

The mean and individual Tc during the HWI for each condition is illustrated in Figure 2, and the trends in individual core temperature during hot-water immersion across conditions are illustrated in Figure 3. Due to technical problems (the capsule could not connect to the data logger in an 8H trial and we speculate that it had passed), Tc was missing for one participant, and therefore, data were analyzed for 11 participants. The mean HWI Tc was significantly lower in 1H compared to 10M (−0.42 ± 0.33°C; *p* = 0.0032; ES = 1.70, ±0.72) and 8H (−0.39 ± 0.37°C; *p* = 0.0140; ES = 1.11, ±0.57). There was no significant difference for mean HWI Tc between 10M and 8H (−0.04 ± 0.20°C; *p* = 0.8842, ES = −0.14, ±0.43). The peak HWI Tc was significantly higher in 8H compared to 1H (0.49 ± 0.42°C, *p* = 0.003, ES = 1.25, ±0.74), but there were no significant differences for peak HWI Tc between the other conditions (*p* > 0.05).

**Figure 4:**
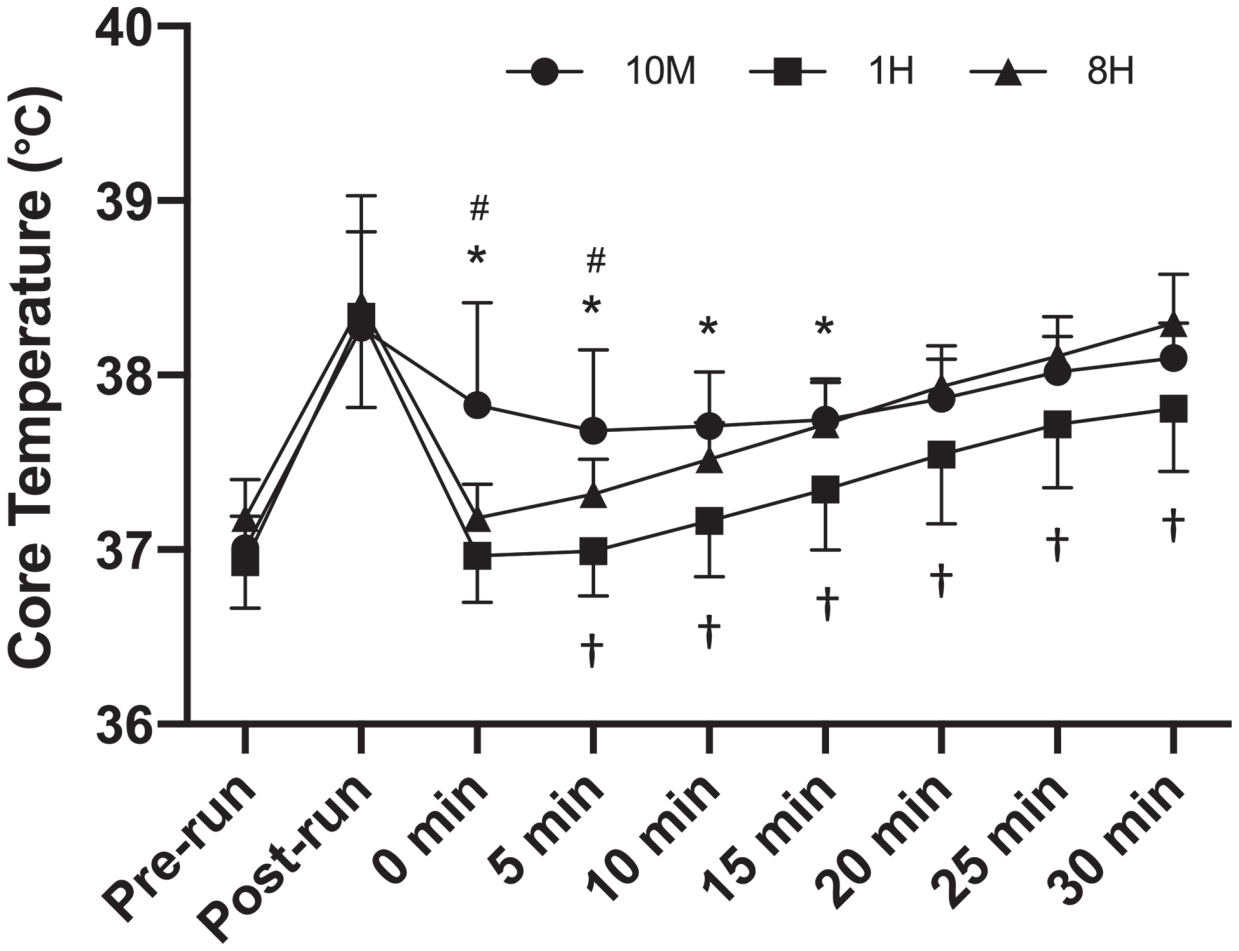
Time course of core temperature changes across the trials for each condition; 10 min (10M), 1 h (1H) and 8 h (8H) intervals (*n* = 11). ^*^10M sig different to 1H, ^#^10M sig different to 8H, ^†^1H sig different to 8H.

The time course of the Tc responses throughout the trials is illustrated in Figure 4. The 10M condition resulted in the highest Tc responses initially, which were significantly higher than 1H from 0 to 15 min (*p* = 0.0008–0.0163; ES = 1.06–2.95) and 8H from 0 to 5 min (*p* = 0.0099–0.0449; ES = 1.67–3.03). The Tc in the 8H condition was significantly higher than the 1H condition from 5 to 30 min (*p* = 0.0079–0.0399; ES = 0.91–1.25).

**Figure 5:**
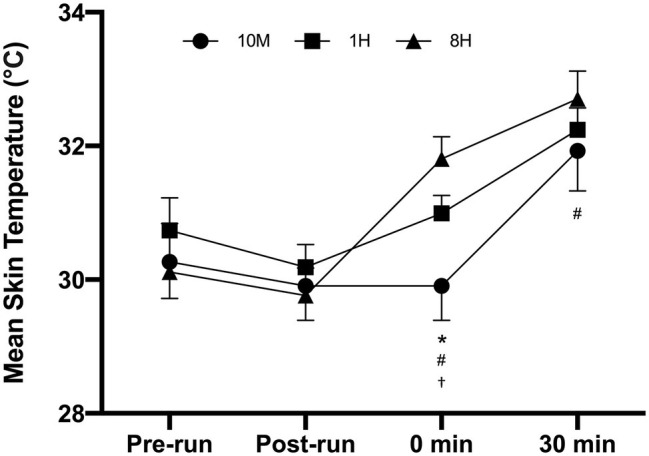
Time course of mean skin temperature changes across the trials for each condition; 10 min (10 M), 1 h (1H), and 8 h (8H) intervals (*n* = 10). ^*^10M sig different to 1H, ^#^10M sig different to 8H, ^†^1H sig different to 8H.

The time course of the Tsk responses throughout the trials is illustrated in Figure 5. Due to technical problems, data were missing for two participants, and therefore, data were analyzed for 10 participants. Upon commencing the HWI, 8H resulted in significantly higher Tsk than both 10M (*p* < 0.0001; ES = 3.27, ±1.52) and 1H (*p* = 0.0013; ES = 2.74, ±1.44). The Tsk was also significantly higher for 1H compared to 10M at 0 min (*p* = 0.0002; ES = 1.90, ±0.92). At the end of the HWI, 8H remained significantly higher than 10M (*p* = 0.0042; ES = 1.19, ±0.68), but there were no significant differences between the other conditions.

A summary of the heart rate, sweat and perceptual responses during the HWI with effect size comparisons is illustrated in Table 1. Due to technical problems, heart rate data were missing for one participant during 8H and therefore data for 11 participants were analyzed. The mean heart rate response was significantly higher in 10M compared to 1H (36 ± 21 bpm; *p* = 0.0006) and 8H (45 ± 18 bpm; *p* < 0.0001). The maximum heart rate response was also significantly higher in 10M compared 1H (41 ± 24 bpm; *p* = 0.0005) and 8H (52 ± 21 bpm; *p* < 0.0001). There were no other significant differences between conditions for any other variable.

**Table 1:** Summary of the heart rate, sweat, and perceptual responses during hot-water immersion with effect size comparisons.

	10M (mean ± SD)	1H (mean ± SD)	8H (mean ± SD)	10M–1H (ES, ±CI)	10M–8H (ES, ±CI)	1H–8H (ES, ± CI)
Mean HR (bpm)	130 ± 19	94 ± 22^*^	85 ± 11^*^	1.35, ±0.46	2.81, ±0.59	0.61, ±0.45
Max HR (bpm)	143 ± 21	102 ± 24^*^	91 ± 13^*^	1.39, ±0.47	2.77, ±0.60	0.60, ±0.44
SL (ml)	700 ± 376	482 ± 259	665 ± 244	0.51, ±0.68	−0.14, ±0.87	−1.05, ±0.81
SR (ml^.^min^.^kg^−1^)	0.29 ± 0.16	0.20 ± 0.10	0.28 ± 0.12	0.53, ±0.70	−0.13, ±0.82	−0.97, ±0.76
Mean TC (AU)	1.8 ± 0.5	1.9 ± 0.5	2.0 ± 0.5	−0.19, ±0.49	−0.43, ±0.63	−0.22, ±0.65
Mean TS (AU)	5.1 ± 0.6	5.2 ± 0.9	5.4 ± 0.7	−0.01, ±0.24	−0.36, ±0.28	−0.35, ±0.39

*10M, 10 min delay; 1H, 1 h delay; 8H, 8 h delay; AU, arbitrary units; bpm, beats per minute; CI, 90% confidence interval; ES, effect size; SD, standard deviation; HR, heart rate; TC, thermal comfort; TS, thermal sensation; SL, sweat loss; SR, sweat rate*.

* *Significantly different to 10M*.

## Discussion

The major finding of the current investigation was that a significantly lower Tc response was measured during HWI following a 1 h delay compared to a 10 min delay (−0.42°C), between exercise and immersion (Figure 2). Further, mean heart rate was also lower following the 1 h delay, compared to the 10 min delay (−36 bpm). Therefore, within the conditions of the current protocol, we partially accept the hypothesis that delaying HWI by 1 h does reduce acute markers of thermo-physiological strain during a post-exercise HWI session. The second major finding was that, within the conditions of the current protocol, the Tc responses were similar between a 10 min and an 8 h delay between exercise and immersion (0.04°C; Figure 2), and peak HWI Tc was greatest in the 8H condition, likely due to a circadian rhythm influence on Tc.

As per Figure 3, the individual participants responded similarly to the HWI between trials (i.e., those with the lowest Tc in one trial generally had the lowest Tc in the others), which may be partly explained by individual anthropometrical characteristics. There were differences in the time course of Tc changes throughout the HWI between trials (Figure 4). During 10M, the group mean Tc remained stable for the first 15 min of immersion (37.7–37.8°C), before increasing to 38.1°C in the final 10 min. We speculate that the prior exercise and subsequent increased core temperature at immersion onset suppressed the rise in Tc in 10M. For 1H and 8H, pre-immersion Tc was significantly lower prior to immersion compared to 10M due to additional recovery after the exercise (see Figure 4). Hence, the Tc profile increased in a linear fashion in these trials, albeit from a higher starting value in 8H, which was the trial with the highest peak Tc at the end of immersion (38.3°C). Considering that the Tc group mean only surpassed 38°C in the final 10 min of immersion in 10M and 8H (and not at all in 1H), the HA potential was likely low for this specific HWI strategy (i.e., 39°C for 30 min to the level of the waist). It should also be noted that athletes may regularly perform exercise at higher intensities than that prescribed in the current study, which may increase the thermo-physiological responses presented.

The HWI strategy presented in the current study (39°C for 30 min to the level of the waist) was deemed a suitable initial exposure based on piloting, but the strategy should become more aggressive (i.e., increased temperature, duration and/or depth) over time to induce a greater rise in Tc and a more sufficient thermal stimulus for adaptation (which also needs to be maintained) to increase the likelihood of inducing meaningful heat adaptations. A previous investigation on post-exercise HWI that successfully induced heat adaptation and performance improvement in runners, implemented a HWI strategy of 40°C for 40 min to the level of the neck (Zurawlew et al., 2016). However, 6/10 participants could not complete this protocol on the first exposure, and therefore, it represents a starting point that is too challenging for many individuals. With this protocol, the researchers demonstrated that core body temperature was increased on average by 1°C throughout the immersion period (following the exercise), across six exposures. In comparison, the current study did not observe such an increase and instead participants completed immersion with a similar core temperature to that observed at the end of the run. Hence, athletes using this technique should aim to quickly increase the demands of the HWI toward 40°C for 40 min to the level of the neck in subsequent exposures as appropriate for the individual. Immersed athletes should be given clear instructions to discontinue HWI when they feel uncomfortably hot or experience any symptoms of pre-syncope or heat illness (i.e., cramping, vomiting, nausea, severe headache, and collapse/fainting). It is also advisable to measure Tc in order to ensure the HWI protocol is both safe and appropriate.

The 8H condition resulted in the highest peak Tc (i.e., at the end of immersion), and a similar mean Tc during HWI compared to 10M. This was somewhat unexpected but may be explained by the higher circadian Tc that occurs in the afternoon (Słomko and Zalewski, 2016). As per Figures 4, 5, there were higher pre-immersion Tc and Tsk in 8H compared to 1H, which does suggest a circadian influence. Considering that all trials commenced at a similar time of day (6:00–7:30 am), this meant that the immersion in the 8H trial always commenced between 2:30 and 4:00 pm; a time consistent with the time of day (3:00 and 5:00 pm) that peak circadian rhythm Tc occurs (Słomko and Zalewski, 2016). Hence, the current data suggest that performing HWI during this time is more effective at increasing acute Tc than HWI performed in the morning when there is a delay of at least 1 h between exercise and immersion, and importantly, HA does not appear to be time of day dependent (Zurawlew et al., 2018b). However, performing HWI at this time should be tested within a longer-term heat adaptation study before such recommendations are made explicit for athletes for heat acclimation purposes. Finally, if the HWI is to be conducted in the afternoon, then it may also be beneficial to conduct the training session at this time as well.

Both the mean and maximal heart rates were significantly increased during HWI in 10M compared to both 1H and 8H. It is likely that the 10M condition did not allow for complete heart rate recovery after the exercise, prior to the immersion, and as such, the participants were subject to increased cardiovascular strain during the HWI in 10M. No significant differences were observed for the sweat responses or the perceptions of thermal comfort and sensation. However, effect size statistics revealed a moderate increase in sweat loss and rate in 8H compared to 1H. It was surprising that there were no differences in thermal perception despite differences in both core temperature and skin temperature, which play a large role in modulating these thermal perceptions. The other interesting finding was that Tsk was significantly higher in 1H compared to 10M before immersion (see Figure 5), which may be explained by the convection and evaporation load associated with running, and/or the additional clothing worn by participants during the 1 h delay in 1H (despite the use of a short stabilization period).

The primary objective of the current study design was to maximize ecological validity to provide clear guidelines for athletes on the timing of post-exercise HWI when it is to be implemented outside of the laboratory setting. Indeed, the availability of a hot-bath immediately after training (i.e., within a few minutes) is not practical for most athletes, but this has not been considered previously. Post-exercise HWI that is slightly delayed after a training session (i.e., 10 min or longer) is practical where the athlete has access to a bath at home. The different time delays chosen in the current study reflected likely delays to occur in the field, but investigation into other time delays is also warranted, especially delays of between 20 and 50 min, within which there is likely a threshold where the thermo-physiological response to HWI is reduced, decreasing the potential capacity for HA. Future research could also investigate the effects of post-exercise HWI when exercise is performed in the afternoon, in hotter environments, or after exercise in additional clothing (Stevens et al., 2017). Considering the ecological design, the current study’s strengths can also be considered as limitations, for example, the athletes drank to thirst during the exercise, ate their usual diet, and completed their usual activities throughout the day instead of remaining in the laboratory during the delays in the 1H and 8H trials. As such, hydration, the thermic effect of food, and incidental physical activity, which can all contribute to heat storage, were not highly standardized. Fluid ingestion was not measured between exercise and HWI and hydration status was not measured either, but the participants were encouraged to drink during and after the exercise, and there was no difference in measures of body mass between exercise endpoint and starting HWI. We also highlight that this study is only an acute study of the physiological and perceptual responses to the different time delays between exercise and HWI, and more long-term studies are needed to determine any effects on heat adaptation.

It should also be noted that in the 8H trial, the ingestible capsule was in the gastrointestinal tract for an additional 8 h compared to the 10M trial and possibly moved further along the tract. However, this is unlikely to have affected the core temperature observations as previous research determined no differences between measures of core body temperature by rectal probe and ingestible capsule at 1 h (0.15 ± 1 0.11°C) vs. 36 h (0.15 ± 0.14°C), after ingestion (Ducharme et al., 2001). Another study has demonstrated some small gastrointestinal temperature gradients, but the most significant gradient was between the stomach and the small intestine (0.2–0.3°C), and any other gradients were trivial (Kolka et al., 1993). We implemented an 8-h timeframe between ingestion and the first measurement, exceeding the 6-h recommendation between ingestion and measurement to ensure that the capsule clears the stomach (Byrne and Lim, 2007), minimizing the effects of any gastrointestinal temperature gradient.

Overall, the current study provides new recommendations for athletes aiming to maximize the acute thermo-physiological response to post-exercise HWI. Immersion should commence immediately after training (within 10 min) to maximize acute Tc and heart rate responses. If this is not viable, an alternative approach may be to implement HWI in the afternoon when Tc is naturally elevated due to circadian rhythm. In the current design, delays of 1 h between exercise and immersion result in significantly lower Tc responses compared to delays of 10 min and 8 h, and Tc of less than 38°C throughout the whole immersion period (when considering the group mean), and hence, a 1 h delay is not recommended for athletes aiming to maximize the acute thermo-physiological response to post-exercise HWI.

## Data Availability Statement

The datasets generated for this study are available on request to the corresponding author.

## Ethics Statement

The studies involving human participants were reviewed and approved by the Southern Cross University Human Research Ethics Committee. The patients/participants provided their written informed consent to participate in this study.

## Author Contributions

SH collected the data. CS and SH wrote the manuscript. SH, PH, SZ, LT, and CS contributed to the study design and performed critical revisions of the manuscript.

### Conflict of Interest

The authors declare that the research was conducted in the absence of any commercial or financial relationships that could be construed as a potential conflict of interest.
